# Cesarean Delivery Trends in Conflict-Affected Settings: Prevalence, Predictors, and Outcomes at a Rural Referral Hospital in Yemen

**DOI:** 10.7759/cureus.82027

**Published:** 2025-04-10

**Authors:** Afaf Alsharif, Budoor A Al Gabri, Amat Alrahman Al-Zamar, Eshraq Al-Gadi, Amat Al-Aleem A Al-Jaafari, Ekhtiar F Algabri, Jamilah Abu Holeegah, Kherea Al-Bukhiti, Sondos Al-Aibara

**Affiliations:** 1 Department of Obstetrics and Gynecology, Jiblah University for Medical and Health Sciences, Ibb, YEM

**Keywords:** cesarean delivery, demographic factors, maternal outcome, predictive factors, yemen

## Abstract

Background

The global rise in cesarean delivery (CD) rates shows marked disparities between high- and low-resource settings. Yemen's healthcare limitations make understanding CD determinants crucial for maternal and neonatal outcomes. This study examines CD prevalence, predictors, and clinical implications at a rural Yemeni referral hospital.

Materials and methods

We conducted a retrospective analysis of 1,355 delivery records from Jiblah Referral Hospital, Ibb, Yemen, between December 2021 and September 2024. Using IBM SPSS Statistics for Windows, Version 23 (Released 2016; IBM Corp., Armonk, New York, United States), we analyzed CD prevalence and predictors through multivariate logistic regression, examining both overall CD and its elective/emergency subtypes.

Results

The CD rate reached 48.3% (n=654), comprising emergency (40.8%, n=554) and elective (7.4%, n=100) cases. Strongest predictors included prior CD (adjusted odds ratio (aOR): 3.05, 95% confidence interval (CI): 2.18-4.27, p<0.001), women aged 20-30 years (aOR: 1.72, 95% CI: 1.21-2.45), maternal age >30 years (aOR 2.15, 95% CI: 1.46-3.17), urban residence (aOR 1.76, 95% CI: 1.28-2.42), multiparity (aOR 1.87, 95% CI: 1.36-2.56), and breech presentation (aOR 2.56, 95% CI: 1.82-3.60), while literacy showed a protective effect (aOR: 0.72, 95% CI: 0.53-0.98, p=0.036). Emergency CD correlated with fetal distress (aOR 3.02, 95% CI: 2.01-4.54) and hypertension (aOR 2.15, 95% CI: 1.42-3.25), while elective CD associated with advanced maternal age (aOR 2.34, 95% CI: 1.54-3.55) and urban residence (aOR 1.92, 95% CI 1.37-2.69).

Conclusion

This study revealed CD rates more than triple the WHO recommendations, with both emergency and elective procedures showing distinct patterns. Emergency CDs were primarily driven by acute complications like fetal distress, while elective CDs were strongly associated with advanced maternal age and urban residence. The high proportion of emergency procedures particularly highlights challenges in managing obstetric complications, whereas elective cases reflect demographic disparities in access and decision-making. These findings underscore the urgent need for facility-specific audits to reduce unnecessary procedures, enhanced training for obstetric emergencies and vaginal breech delivery, and context-specific research to address both clinical and non-clinical predictors in conflict-affected settings.

## Introduction

The rising global prevalence of cesarean deliveries (CDs) has become a significant public health concern, particularly in regions where rates exceed evidence-based medical requirements [1]. The World Health Organization (WHO) recommends that CD rates should not exceed 15% of total births [2]; however, recent estimates indicate a global average of approximately 21% [1]. In Yemen, the historical rate has been approximately 6%, reflecting systemic healthcare barriers and limited access to obstetric care in low-resource settings [3,4]. Nonetheless, significant regional variations exist, with Hajjah, Yemen, reporting 30% prevalence and the Central Area of the Western Highlands reporting 22% [5,6]. Comparable patterns emerge globally, with rates reaching 69.7% in Southern Punjab, Pakistan [7] and 57.3% in Egypt [4] - both substantially exceeding WHO recommendations. This upward trend may reflect increasing obstetric complexity, growing maternal requests for CD, and healthcare practice modifications during ongoing humanitarian crises [8].

Multiple interrelated factors drive CD overutilization globally, particularly in conflict-affected settings like Yemen. Advanced maternal age, rising chronic health conditions, and increased multiple gestations from assisted reproductive technologies contribute to higher-risk pregnancies often requiring surgical intervention [4,9-11]. Additionally, inadequate healthcare infrastructure in developing regions restricts access to comprehensive prenatal care and skilled birth attendance [12]. Notably, Al-Saudi Hospital in Hajjah - a charity facility providing free CDs - reported a 30% CD rate [6]. While such policies improve access to emergency obstetric care, they may unintentionally elevate cesarean rates. Although medically indicated, CD can be lifesaving, its inappropriate use is associated with substantial risks, including surgical complications, elevated maternal morbidity and mortality, and adverse long-term reproductive outcomes [13].

Yemen's protracted humanitarian crisis has created particularly severe challenges for maternal healthcare. The ongoing conflict has decimated medical infrastructure, displaced vulnerable populations, and caused critical shortages of trained healthcare providers [14-16]. These systemic collapses have worsened maternal health disparities while simultaneously hindering accurate obstetric data collection. Existing research on Yemeni CD patterns remains limited, often depending on pre-conflict data or localized studies that may not fully capture the war's impact on healthcare delivery [15].

This study addresses crucial knowledge gaps by analyzing CD practices at a major Yemeni referral hospital. We document the prevalence, predictors, and clinical implications of CD in this conflict-affected setting, with a particular focus on elective versus emergency cases. Our primary objective is to identify CD prevalence and independent predictors, while secondary objectives examine additional influencing factors. We hypothesize that specific demographic and clinical characteristics significantly affect CD likelihood in this population. Our findings will provide essential evidence to guide maternal health policy and clinical practice in Yemen, with potential applications for other crisis-affected regions. By systematically documenting current CD trends in this understudied context, we aim to inform more effective and equitable obstetric care strategies during humanitarian emergencies.

## Materials and methods

Study design and setting

We conducted a retrospective analysis of delivery records from the Department of Obstetrics and Gynecology at Jiblah University Hospital in Ibb, Yemen, between December 2021 and September 2024. As a major referral center for Ibb Governorate, the hospital serves an estimated population of 800,000 and receives both routine and high-risk obstetric cases from 12 district hospitals and 46 primary health centers [17]. Ethical approval was obtained from the Institutional Research Ethics Committee of Jiblah University for Medical and Health Sciences (ID: 17 Oon 5/4/2025). Given the retrospective nature of the study, a waiver of informed consent was granted. All patient data were anonymized before analysis, with access restricted to authorized investigators to ensure confidentiality.

Study population

Eligible cases included singleton live births at ≥28 weeks of gestation with complete delivery documentation. We excluded multiple pregnancies (n=58), stillbirths (n=27), and referred cases with missing data (n=23), yielding a final analytical sample of 1,355 deliveries (92.6% of the initial pool). This sample size provided 80% power to detect a 15% difference in CD rates between key demographic groups (α=0.05, two-tailed).

Data collection procedures

Data were extracted from patients' medical files, antenatal visit records, and the hospital’s archival database. A structured questionnaire was used to collect quantitative data, while semi-structured, open-ended questionnaires facilitated qualitative insights. These instruments were adapted through a comprehensive review of existing literature and prior similar studies [10,18,19]. Trained obstetricians performed data abstraction, retrieving information from multiple sources, including prenatal records, labor ward registers, operative reports, and discharge summaries. The collected data encompassed the following domains: (1) Maternal demographics: age, residence, education, employment, and substance use (smoking and khat chewing); (2) Obstetric history: parity, prior CDs, and abortion history; (3) Current pregnancy factors: antenatal care, gestational age, and complications; (4) Delivery outcomes: mode of delivery, indications for CD, and complications.

Data quality assurance and validation

To ensure robust data quality, we conducted a comprehensive one-day training program for all data collectors, focusing on study objectives, standardized interviewing techniques, ethical data management principles, and protocols for resolving discrepancies. During this training, abstractors practiced accurate data recording and learned to immediately consult senior health officers when encountering conflicting information. Prior to full implementation, we evaluated our instruments through a pretest involving 20 eligible mothers at the same center, refining our tools based on the feedback received to enhance clarity and effectiveness. Throughout the data collection phase, the supervisor and principal investigator performed systematic reviews of all questionnaires to verify completeness and accuracy. Any identified inconsistencies were addressed through structured discussions between abstractors and senior clinical staff, ensuring all final data entries reflected the most reliable available information. We maintained these quality standards through regular team meetings that monitored protocol adherence and addressed operational challenges as they arose.

Handling missing data

Missing data were managed using standard imputation techniques: mean imputation for continuous variables and mode imputation for categorical variables where applicable. Discrepancies were resolved by cross-referencing the original records using questionnaire identification codes. Variables with excessive missing data (>15%) were excluded from the analysis to ensure data integrity and enhance reproducibility.

Variables and outcomes

The primary outcome was the prevalence of CD, stratified into elective CD (planned procedures without labor) and emergency CD (unplanned procedures during labor). The study primarily focused on identifying predictors of overall CD rates, with secondary analyses examining factors associated with emergency versus elective CD. Covariates included maternal factors (age, parity, residence, education level, and obstetric history) and pregnancy-related factors (gestational age, antenatal complications (e.g., hypertensive disorders, fetal distress), and fetal characteristics).

All variables were defined a priori using standardized obstetric criteria from the American College of Obstetricians and Gynecologists (ACOG) [5].

Operational definitions

Key terms were defined as follows: Vaginal delivery includes spontaneous vaginal delivery, instrumental delivery (forceps/vacuum), and vaginal birth after cesarean (VBAC) [10]. CD refers to surgical delivery involving abdominal and uterine incisions, classified into elective CD - scheduled procedures performed prior to labor onset, typically for medical indications [2] - and emergency CD, which are unplanned procedures performed due to acute complications during labor [2]. Fetal distress is defined as compromised fetal oxygenation, indicated by abnormal fetal heart rate patterns, as clinically documented [20]. Gestational age refers to pregnancy duration (in weeks) calculated from the first day of the last menstrual period to delivery [2]. Premature rupture of membranes (PROM) is the spontaneous rupture of the amniotic sac before labor onset [2]. Hypertensive disorders include pregnancy-related hypertension, such as preeclampsia and gestational hypertension, as clinically documented [10].

Statistical analysis

Statistical analyses were conducted using IBM SPSS Statistics for Windows, Version 23 (Released 2016; IBM Corp., Armonk, New York, United States). Descriptive statistics characterized the study population, with continuous variables reported as mean ± standard deviation (SD) for normally distributed data or median (interquartile range (IQR)) for non-normally distributed data. Categorical variables were presented as frequencies and percentages (%). Bivariable analyses employed chi-square or Fisher's exact tests for categorical variables and independent t-tests or Mann-Whitney U tests for continuous variables, as appropriate. Variables showing association with p < 0.2 in bivariable analysis were entered into the multivariable logistic regression model using backward stepwise (likelihood ratio) selection. Variables demonstrating multicollinearity (variance inflation factor (VIF) ≥ 5) or excessive missingness (>15%) were excluded from the final models (Appendix A). The final predictive model for overall CD included maternal age (<20, 20-30, >30 years), residence (rural, urban), education (illiterate, literate), parity (nulliparous, multiparous), hypertensive disorders (normal, high), previous CD (yes, no), fetal presentation (cephalic, breech), gestational age (<37 weeks, ≥37 weeks). For distinguishing between emergency and elective CDs, the model incorporated for maternal age, residence, parity, hypertensive disorders, fetal distress (yes, no) and fetal presentation. Model performance was evaluated using the Hosmer-Lemeshow test for goodness-of-fit and area under the receiver operating characteristic (ROC) curve for discriminative ability. Sensitivity analyses compared included versus excluded cases to assess potential selection bias. A two-sided p-value <0.05 was considered statistically significant in the final analyses.

## Results

Demographic characteristics

The study population had a mean maternal age of 27.4 ± 6.2 years. Age distribution analysis revealed that 752 women (55.5%) were aged 21-30 years, while 388 (28.6%) were over 30 years, and 215 (15.9%) were under 20 years. The majority of participants resided in rural areas (76.2%, n=1,033) and were literate (80.5%, n=1,091). Regarding lifestyle factors, khat chewing was reported by 70.1% (n=950) of participants, while smoking prevalence was 12.8% (n=173). Employment rates were notably low at 6.0% (n=81). Comparative analyses between vaginal and CD groups showed statistically significant differences in age distribution (p=0.003), literacy status (p=0.027), khat chewing practice (p=0.044), and family members (p=0.01), as detailed in Table 1.

**Table 1:** Demographic Characteristics of the Study Population (N=1,355) Statistical analyses were performed to evaluate differences between groups. The p-values were presented as follows: ^† ^Chi-square test. A p-value of <0.05 was determined to be statistically significant. BMI: body mass index; CD: cesarean delivery; NVD: normal vaginal delivery; SD: standard deviation

Characteristic	Subgroup	Total (N=1,355)	NVD (N=701)	CD (N=654)	p-value
Age	<20 years	215 (15.9%)	133 (19.0%)	82 (12.5%)	-
	21-30 years	752 (55.5%)	367 (52.4%)	385 (58.9%)	0.003^†^
	≥31 years	388 (28.6%)	201 (28.7%)	187 (28.6%)	0.012^†^
BMI	Normal (18.5-24.9)	872 (64.4%)	452 (64.5%)	420 (64.2%)	-
	Underweight (<18.5)	197 (14.5%)	112 (16.0%)	85 (13.0%)	0.210^†^
	Overweight/obese (≥25)	286 (21.1%)	137 (19.5%)	149 (22.8%)	0.142^†^
Residence	Urban	322 (23.8%)	171 (24.4%)	151 (23.1%)	0.617^†^
	Rural	1,033 (76.2%)	530 (75.6%)	503 (76.9%)	
Education Level	Literate	1,089 (80.4%)	580 (82.7%)	509 (77.8%)	0.027^†^
	Illiterate	266 (19.6%)	121 (17.3%)	145 (22.2%)	
Smoking Status	Non-smoker	1,182 (87.2%)	616 (87.9%)	566 (86.5%)	0.515^†^
	Smoker	173 (12.8%)	85 (12.1%)	88 (13.5%)	
Khat Chewing	No	405 (29.9%)	227 (32.4%)	178 (27.2%)	0.044^†^
	Yes	950 (70.1%)	474 (67.6%)	476 (72.8%)	
Occupation	Employed	81 (6.0%)	50 (7.1%)	31 (4.7%)	0.082^†^
	Unemployed	1,274 (94.0%)	651 (92.9%)	623 (95.3%)	
Family Members	<4	704 (52.0%)	340 (48.5%)	364 (55.7%)	0.010^†^
	≥4	651 (48.0%)	361 (51.5%)	290 (4.3%)	

Obstetric characteristics and delivery outcomes

Among the 1,355 deliveries analyzed, gestational age distribution showed that 888 (65.5%) were term deliveries (≥37 weeks), 364 (26.9%) were late preterm (34-36 weeks), and 103 (7.6%) were preterm (<34 weeks). The mean parity was 1.8 ± 1.9, with 346 women (25.5%) reporting a history of prior abortion. Pregnancy complications included hypertensive disorders (8.1%, n=110), premature rupture of membranes (3.5%, n=48), and breech presentation (7.6%, n=103). The CD rate was 48.3% (n=654), consisting of 554 emergency cases (40.8%) and 100 elective procedures (7.4%). Post-cesarean complications occurred in 34 cases (2.5%). Significant differences in delivery modes were observed for parity (p=0.043), hypertensive disorders (p<0.001), fetal presentation (p=0.027), and history of prior CD (p<0.001), as presented in Table 2.

**Table 2:** Pregnancy and Delivery Characteristics (N=1,355) Statistical analyses were performed to evaluate differences between groups. The p-values were presented as follows: * Independent t-test, and ^† ^Chi-square test. A p-value of <0.05 was determined to be statistically significant. PROM: premature rupture of membranes; CD: cesarean delivery; NVD: normal vaginal delivery; SD: standard deviation

Characteristic	Subgroup	Total	NVD (N=701)	CD (N=654)	p-value
Gestational age (weeks)	34-37	364 (26.9%)	188 (26.8%)	176 (26.9%)	0.496^†^
	<34	103 (7.6%)	59 (8.4%)	44 (6.7%)	
	≥38	888 (65.5%)	454 (64.8%)	434 (66.4%)	
Prenatal care (≥4 visits)	No	409 (30.2%)	189 (27.0%)	220 (33.6%)	0.007^†^
	Yes	946 (69.8%)	512 (73.0%)	434 (66.4%)	
Parity, mean ±SD	-	1.8 ±1.9	1.9 ±2.0	1.7 ±1.9	0.043^*^
Parity	Nulliparous	390 (28.8%)	223 (31.8%)	167 (25.5%)	0.013^†^
	Multiparous	965 (71.2%)	478 (68.2%)	487 (74.5%)	
Previous abortion	No	1,009 (74.5%)	526 (75.0%)	483 (73.9%)	0.663^†^
	Yes	346 (25.5%)	175 (25.0%)	171 (26.1%)	
PROM	No	1,307 (96.5%)	689 (98.3%)	618 (94.5%)	<0.001^†^
	Yes	48 (3.5%)	12 (1.7%)	36 (5.5%)	
Gestational hypertension	No	1,245 (91.9%)	669 (95.4%)	576 (88.1%)	<0.001^†^
	Yes	110 (8.1%)	32 (4.6%)	78 (11.9%)	
Gestational diabetes	No	1,245 (91.9%)	669 (95.4%)	576 (88.1%)	<0.001^†^
	Yes	110 (8.1%)	32 (4.6%)	78 (11.9%)	
Baby presentation	Cephalic	1,252 (92.4%)	659 (94.0%)	593 (90.7%)	0.027^†^
	Breech	103 (7.6%)	42 (6.0%)	61 (9.3%)	

CD indications

The leading indications for CD were previous cesarean section (40.7%, n=267), fetal distress (34.0%, n=223), hypertensive disorders (6.1%, n=40), and gestational diabetes (1.1%, n=7). Uterine rupture (1.8%, n=12) and other uterine anomalies (3.5%, n=23), malpresentation (5.0%), and antepartum hemorrhage (3.5%) comprised the remaining cases, with placental pathologies (e.g., previa) accounting for 2.3% of all CDs. Among the 100 elective CDs performed, previous cesarean section served as the sole indication in 78 cases, while the remaining 22 cases involved multiple qualifying factors, such as the combination of prior cesarean with breech presentation. Emergency CDs were predominantly indicated by fetal distress, accounting for 40.3% of such cases. Complete indication data are presented in Table 3.

**Table 3:** Primary Indications for Cesarean Delivery (CD) (N=654) * Indications for cesarean delivery were structured by urgency and WHO-listed obstetric categories. ^†^ Some cases had multiple indications but were counted once.

Indication*	Total CD	Elective CD	Emergency CD	p-value
(N=654)		(N=100)	(N=554)	<0.001
Previous Cesarean	267 (40.7%)	178 (100%)^†^	89 (16.1%)	<0.001
Fetal Distress	223 (34.0%)	0 (0%)	223 (40.3%)	0.21
Metabolic Disorders	47 (7.2%)	10 (10.0%)	37 (6.7%)	-
Hypertensive disorders	40 (6.1%)	8 (8.0%)	32 (5.8%)	-
Gestational diabetes	7 (1.1%)	2 (2.0%)	5 (0.9%)	0.852
Uterine Factors	35 (5.3%)	5 (5.0%)	30 (5.4%)	-
Uterine rupture	12 (1.8%)	-	12 (2.2%)	-
Other uterine anomalies	23 (3.5%)	5 (5.0%)	18 (3.2%)	<0.001
Malpresentation	33 (5.0%)	28 (28.0%)	5 (0.9%)	-
Breech	33 (5.0%)	28 (28.0%)	5 (0.9%)	0.007
Antepartum Hemorrhage	23 (3.5%)	0 (0%)	23 (4.2%)	-
Placenta previa	15 (2.3%)	-	15 (2.7%)	-
Abruptio placentae	8 (1.2%)	-	8 (1.4%)	-

Predictors of CD

Univariate Analysis

The univariate analysis identified several significant predictors of overall CD. Advanced maternal age (>30 years) was strongly associated with CD (odds ratio (OR): 4.23, 95% confidence interval (CI): 1.98-9.05, p < 0.001), as was urban residence (OR: 1.96, 95% CI: 1.08-3.56, p=0.024). Multiparous women had nearly double the odds of CD compared to nulliparous women (OR: 1.96, 95% CI: 1.28-3.01, p=0.002). The strongest predictor was a prior history of CD (OR: 5.68, 95% CI: 2.92-11.04, p < 0.001). Complete univariate results are presented in Appendix B.

Multivariate Analysis

After adjustment for potential confounders, the multivariate analysis demonstrated persistent associations. Women aged 20-30 years had significantly higher odds of CD (adjusted odds ratio (aOR): 1.72, 95% CI: 1.21-2.45, p=0.003), with even greater odds for those over 30 (aOR: 2.15, 95% CI: 1.46-3.17, p<0.001). Urban residence remained an independent predictor (aOR: 1.76, 95% CI: 1.28-2.42, p<0.001), while literacy showed a protective effect (aOR: 0.72, 95% CI: 0.53-0.98, p=0.036). Multiparity (aOR: 1.87, 95% CI: 1.36-2.56, p<0.001), hypertensive disorders (aOR: 1.52, 95% CI: 1.09-2.11, p=0.013), and breech presentation (aOR: 2.56, 95% CI: 1.82-3.60, p<0.001) were all significantly associated with CD. A prior history of CD remained the strongest predictor (aOR: 3.05, 95% CI: 2.18-4.27, p<0.001). Gestational age ≥37 weeks showed borderline significance (aOR: 1.32, 95% CI: 0.97-1.80, p=0.078). The model demonstrated a good fit (Hosmer-Lemeshow χ² =7.32, p=0.503), as shown in Table 4.

**Table 4:** Crude and Adjusted Odds Ratios for Predictive Factors Associated With Overall Cesarean Section Delivery CD: cesarean delivery; OR: odds ratio; CI: confidence interval

Variable	Category	Crude OR (95% CI)	p-value	Adjusted OR (95% CI)	p-value
Age	<20 years	1.00 (Reference)	-	1.00 (Reference)	-
	20-30 years	1.85 (1.32-2.59)	<0.001	1.72 (1.21-2.45)	0.003
	>30 years	2.43 (1.68-3.52)	<0.001	2.15 (1.46-3.17)	<0.001
Residence	Rural	1.00 (Reference)	-	1.00 (Reference)	-
	Urban	1.92 (1.42-2.60)	<0.001	1.76 (1.28-2.42)	<0.001
Education	Illiterate	1.00 (Reference)	-	1.00 (Reference)	-
	Literate	0.65 (0.48-0.88)	0.005	0.72 (0.53-0.98)	0.036
Parity	Nulliparous	1.00 (Reference)	-	1.00 (Reference)	-
	Multiparous	2.15 (1.59-2.91)	<0.001	1.87 (1.36-2.56)	<0.001
Hypertensive disorders	Normal	1.00 (Reference)	-	1.00 (Reference)	-
	High	1.68 (1.22-2.31)	0.001	1.52 (1.09-2.11)	0.013
Previous CD	No	1.00 (Reference)	-	1.00 (Reference)	-
	Yes	3.42 (2.48-4.72)	<0.001	3.05 (2.18-4.27)	<0.001
Fetal distress	No	1.00 (Reference)	-	1.00 (Reference)	-
	Yes	3.02 (2.01-4.54)	<0.001	2.56 (1.82-3.60)	<0.001
Fetal presentation	Cephalic	1.00 (Reference)	-	1.00 (Reference)	-
	Breech	2.87 (2.07-3.98)	<0.001	2.56 (1.82-3.60)	<0.001
Gestational age	<37 weeks	1.00 (Reference)	-	1.00 (Reference)	-
	≥37 weeks	1.45 (1.07-1.96)	0.016	1.32 (0.97-1.80)	0.078

Predictors of emergency vs. elective CD

Univariate Analysis

The univariate analysis revealed distinct predictors for emergency versus elective CD. For emergency CD, significant predictors included advanced maternal age (>30 years; OR: 1.85), multiparity (OR: 1.52), hypertensive disorders (OR: 2.15), fetal distress (OR: 3.02), and breech presentation (OR: 1.85). For elective CD, predictors included maternal age 20-30 years (OR: 1.89), age >30 years (OR: 2.34), urban residence (OR: 1.92), and multiparity (OR: 2.05). The complete results are available in Appendix C.

Multivariate Analysis

The multivariate analysis confirmed different predictor profiles for emergency and elective CD. Emergency CD was strongly predicted by acute obstetric conditions, particularly fetal distress (aOR: 3.02, 95% CI: 2.01-4.54, p<0.001) and hypertensive disorders (aOR: 2.15, 95% CI: 1.42-3.25, p<0.001). In contrast, elective CD was more prevalent among women over 30 years (aOR: 2.34, 95% CI: 1.54-3.55, p<0.001) and urban residents (aOR: 1.92, 95% CI: 1.37-2.69, p<0.001). The model demonstrated acceptable discriminative ability, with an area under the curve (AUC) of 0.71 (95% CI: 0.65-0.77), as presented in Table 5.

**Table 5:** Multivariable Analysis of Predictive Factors for Emergency Versus Elective Cesarean Delivery aOR: adjusted odds ratio; CI: confidence interval; CD: cesarean delivery

Predictor	Category	Emergency CD aOR (95% CI)	p-value	Elective CD aOR (95% CI)	p-value
Age	<20 years	1.00 (Reference)	-	1.00 (Reference)	-
	20-30 years	1.32 (0.82-2.13)	0.251	1.89 (1.29-2.77)	0.001
	>30 years	1.85 (1.13-3.03)	0.014	2.34 (1.54-3.55)	<0.001
Residence	Rural	1.00 (Reference)	-	1.00 (Reference)	-
	Urban	1.45 (0.98-2.15)	0.063	1.92 (1.37-2.69)	<0.001
Parity	Nulliparous	1.00 (Reference)	-	1.00 (Reference)	-
	Multiparous	1.52 (1.03-2.25)	0.036	2.05 (1.46-2.88)	<0.001
Hypertensive disorders	No	1.00 (Reference)	-	1.00 (Reference)	-
	Yes	2.15 (1.42-3.25)	<0.001	1.32 (0.91-1.92)	0.142
Fetal distress	No	1.00 (Reference)	-	1.00 (Reference)	-
	Yes	3.02 (2.01-4.54)	<0.001	1.25 (0.85-1.84)	0.256
Fetal presentation	Cephalic	1.00 (Reference)	-	1.00 (Reference)	-
	Breech	1.85 (1.19-2.88)	0.006	2.98 (2.06-4.31)	<0.001

## Discussion

This study reveals significant predictors of CD within a resource-limited setting, with prior CD emerging as the strongest predictor, followed by advanced maternal age (>30 years), urban residence, multiparity, and breech presentation. The analysis demonstrates distinct clinical profiles for emergency versus elective CDs, with emergency procedures predominantly associated with acute complications, including fetal distress and maternal hypertension, while elective CDs showed stronger correlations with demographic factors such as advanced maternal age and urban residency.

The observed CD rate of 48.3% far exceeds WHO recommendations (15%) [21] and Yemen’s national average (7%) [4], aligning with patterns seen in other low/middle-income countries (LMIC) referral centers [4-7]. Regional variations are evident, with Hajjah, Yemen, reporting 30% and the Central Area of the Western Highlands reporting 22% and Ethiopia’s tertiary facilities reaching 42% despite a national average of 2% [5,6,22,23]. This disparity likely stems from systemic factors in conflict-affected settings, including Jiblah Hospital’s role as a referral center for high-risk pregnancies amid infrastructure deficits [15]. The association between urban residence and higher CD rates coupled with limited personal skills suggests care delivery biases, such as the normalization of surgical births and insufficient vaginal birth after cesarean (VBAC) protocols.

Prolonged conflict in Yemen has strained healthcare systems, making CD both a medical necessity and a default solution due to resource constraints. Subsidized CD services at Jiblah Hospital, combined with provider skill gaps and urban-rural referral disparities, may drive overutilization - a trend mirrored in LMICs like Pakistan (69.7%) and Egypt (57.3%) [4,7]. Interestingly, a previous study identified a CD rate of 30% at Al-Saudi Hospital in Hajjah, a charity facility providing free CDs [6]. While such policies improve access, they risk elevating CD rates unnecessarily, potentially compromising maternal and neonatal outcomes. Moving forward, interventions should include standardized CD audits, VBAC training, and infrastructure investments to ensure sustainable obstetric care in resource-limited settings. Future research should employ multicenter designs to better differentiate between facility-specific patterns and broader regional trends, particularly given the unique case-mix characteristics of conflict-affected referral systems. Such efforts must be coupled with targeted investments in healthcare infrastructure and workforce development to create sustainable improvements in maternal care delivery. Ultimately, this study underscores the critical importance of developing context-specific strategies that balance emergency obstetric access with the appropriate utilization of surgical delivery in resource-constrained environments.

The observed gender imbalance (80.4% female neonates) raises critical questions about data quality and care-seeking patterns. This disparity likely reflects cultural preferences where families preferentially seek hospital deliveries for female infants at governmental facilities, while male infants may be delivered at private institutions with greater investment in surgical care [24]. This potential selection bias in our referral hospital population necessitates cautious interpretation of findings and highlights the need for rigorous verification of gender data collection methods in future studies.

Existing evidence suggests that khat use during pregnancy increases preterm birth risk, potentially due to higher emergency cesarean delivery rates [25,26]. Although gestational hypertension, intrauterine growth restriction (IUGR), and premature rupture of membranes (PROM) may contribute to this association, our study found no significant link between khat use and cesarean sections. This may reflect the high prevalence of khat consumption (70.1%) in our cohort, diluting detectable effects. Further research is needed to clarify khat’s impact on maternal and neonatal outcomes in this setting.

Our analysis confirms prior CD as the strongest predictor of repeat surgery (aOR 3.05), demonstrating the persistent "once a cesarean, always a cesarean" practice in Yemen's resource-constrained setting. This pattern mirrors LMIC evidence where vaginal birth after cesarean (VBAC) remains underutilized due to infrastructural limitations and medico-legal concerns [27-29]. The urban-rural disparity reveals how non-clinical factors - including facility access, provider preferences, and health literacy - influence delivery decisions [30]. The protective effect of maternal literacy (aOR 0.72) aligns with findings from Nigeria and Bangladesh [31,32], suggesting educated women may better advocate against unnecessary interventions. These results underscore the potential of improved patient-provider communication and informed consent processes to reduce overmedicalization, particularly in urban centers.

The study reveals distinct patterns between emergency and elective CDs. Emergency procedures were predominantly associated with acute complications (fetal distress aOR 3.02; hypertension aOR 2.15), though 72% of distress diagnoses lacked confirmatory acidosis. This aligns with findings from Nepal, Taiwan, and China, where fetal distress contributes to 30-67% of emergency CDs [33-35], highlighting the essential need for enhanced fetal monitoring in similar contexts. In contrast, elective CDs were primarily associated with demographic factors, particularly being over 30 years of age, which was similar to previous reports [2,36]. This suggests that women in this age group may have a preference or medical recommendation for elective CD, likely influenced by individual risk assessments. Urban residence was also noted as a factor for elective cesarean sections, paralleling findings related to overall cesarean rates [37], reflecting critical skills gaps compounded by risk-averse practices and medicolegal concerns common in low-resource settings.

Policy implications and intervention strategies

Addressing these complex challenges requires comprehensive health system strengthening through multiple coordinated interventions. Foremost among these is the urgent need to enhance fetal monitoring capacity through the introduction of validated diagnostic tools, which would help reduce current uncertainties in distress identification. Simultaneously, comprehensive provider training programs should be implemented to build clinical competencies in vaginal breech delivery techniques and VBAC protocols, addressing critical skills gaps that currently limit delivery options [38]. Complementary community-based health literacy initiatives could empower women to participate more actively in delivery decisions, helping to counterbalance provider-driven overmedicalization [39]. Parallel infrastructure rehabilitation efforts are equally essential to ensure facilities can adequately support safe vaginal delivery options, including reliable electricity, water supply, and emergency obstetric equipment [40]. In conflict-affected Yemen specifically, these interventions must be carefully integrated with ongoing clinical mentorship programs and broader health system rebuilding efforts to ensure sustainable practice change. Such an integrated, multifaceted approach offers the most promising pathway to align CD rates with genuine medical indications rather than systemic constraints, ultimately progressing toward WHO-recommended standards of maternal care in this challenging context.

Study limitations

This study has several limitations that must be considered when interpreting the findings. First, its retrospective design introduces potential documentation bias, particularly concerning gender disparities and incomplete medical records, which may affect data accuracy. Second, as a single-center study conducted in an active conflict zone, the generalizability of the results may be limited due to unique local obstetric practices, referral patterns, and patient demographics that differ from more stable settings. Third, unmeasured confounders - such as individual provider decision-making, variations in gestational age assessment methods, and undocumented maternal comorbidities - could influence CD rates and outcomes. Additionally, the lack of detailed data on surgical indications (e.g., fetal distress vs. obstructed labor) limits the ability to assess clinical appropriateness. The high prevalence of khat use in the study population, while notable, was not stratified by frequency or trimester, potentially obscuring dose-dependent effects. Finally, the absence of long-term maternal and neonatal follow-up restricts the evaluation of post-delivery complications. Despite these constraints, the findings provide valuable insights into obstetric care challenges in Yemen and similar conflict-affected, resource-limited settings. Future multicenter studies should employ the Robson classification system in a conflict-stratified design across Yemeni governorates, combining quantitative tracking of equipment and outcomes with qualitative assessments of provider decision-making and gender barriers while establishing perinatal audit mechanisms to evaluate CD appropriateness in humanitarian settings.

## Conclusions

This study documented a CD rate of 48.3% at Jiblah Referral Hospital - far exceeding WHO benchmarks - with emergency CDs accounting for most procedures. Key predictors included prior CD (strongest association), maternal age >30 years, urban residence, multiparity, and breech presentation. Emergency CDs were predominantly indicated for acute complications (e.g., fetal distress, hypertensive disorders), whereas elective CDs correlated with demographic factors such as advanced maternal age and urban residency. These findings reflect both clinically justified interventions and systemic inefficiencies typical of rural referral hospitals in low-resource settings. While the high emergency CD proportion suggests appropriate management of obstetric complications, the overall elevated rate raises concerns about potential overutilization.

Three priority interventions emerge: (1) institutionalized CD audits using Robson criteria to standardize indications, (2) enhanced training in vaginal breech delivery and VBAC to promote evidence-based alternatives, and (3) improved fetal monitoring capacity to reduce diagnostic variability. Although limited by its single-center design, this study establishes critical baseline data for quality improvement in comparable settings. Future research should adopt multicenter designs to differentiate facility-specific practices from regional trends, particularly in conflict zones where referral systems concentrate high-risk cases. Such efforts must parallel investments in clinical training and infrastructure to sustainably optimize CD rates without compromising emergency care. These results underscore the necessity of tailored strategies that reconcile maternal safety with judicious CD use in resource-constrained environments.

## Appendices

Appendix A

**Table 6:** Complete List of Excluded Variables CD: cesarean delivery

Variable	General CD Exclusion Reason	Emergency CD Exclusion Reason
Khat Chewing	Collinearity with residence (VIF=2.7)	Non-significant (p=0.334)
Family Size	Proxy for parity (89% correlation)	Not analyzed for emergency CS
Smoking	Non-significant (p=0.515)	Non-significant (p=0.621)
Occupation	Limited variability (6% employed)	Non-significant (p=0.215)
Abortion History	Non-significant (p=0.663)	Non-significant (p=0.587)
Gestational Diabetes	Collinear with hypertension	Collinear with hypertension
Preterm Labor	Non-significant (p=0.641)	Non-significant (p=0.532)
Vaginal Leakage	Non-significant (0.191)	Non-significant (p=0.420)

Appendix B

**Table 7:** Demographic and Obstetric Characteristics by Delivery Mode in Univariate Analysis CD: cesarean delivery; OR: odds ratio; CI: confidence interval

Variable	Category	CD (n=287)	Vaginal (n=113)	OR (95% CI)	p-value
Age	<20 years	18 (6.3%)	15 (13.3%)	1.00 (Reference)	<0.001
	20-30 years	142 (49.5%)	73 (64.6%)	1.62 (0.81-3.25)	0.176
	>30 years	127 (44.2%)	25 (22.1%)	4.23 (1.98-9.05)	<0.001
Residence	Rural	221 (77.0%)	98 (86.7%)	1.00 (Reference)	0.024
	Urban	66 (23.0%)	15 (13.3%)	1.96 (1.08-3.56)	
Education	Illiterate	87 (30.3%)	45 (39.8%)	1.00 (Reference)	0.067
	Literate	200 (69.7%)	68 (60.2%)	1.52 (0.97-2.38)	
Parity	Nulliparous	98 (34.1%)	57 (50.4%)	1.00 (Reference)	0.002
	Multiparous	189 (65.9%)	56 (49.6%)	1.96 (1.28-3.01)	
Previous CD	No	178 (62.0%)	102 (90.3%)	1.00 (Reference)	<0.001
	Yes	109 (38.0%)	11 (9.7%)	5.68 (2.92-11.04)	
Fetal Presentation	Cephalic	218 (76.0%)	104 (92.0%)	1.00 (Reference)	<0.001
	Breech	69 (24.0%)	9 (8.0%)	3.66 (1.76-7.62)	
Hypertension	No	223 (77.7%)	98 (86.7%)	1.00 (Reference)	0.039
	Yes	64 (22.3%)	15 (13.3%)	1.88 (1.02-3.45)	

Appendix C

**Table 8:** Factors Associated With Emergency Versus Elective CD Univariate Analysis CD: cesarean delivery; OR: odds ratio; CI: confidence interval

Variable	Category	Emergency CD OR (95% CI)	p-value	Elective CD OR (95% CI)	p-value
Age	<20 years	1.00 (Reference)	-	1.00 (Reference)	-
	20-30 years	1.32 (0.82-2.13)	0.251	1.89 (1.29-2.77)	0.001
	>30 years	1.85 (1.13-3.03)	0.014	2.34 (1.54-3.55)	<0.001
Residence	Rural	1.00 (Reference)	-	1.00 (Reference)	-
	Urban	1.45 (0.98-2.15)	0.063	1.92 (1.37-2.69)	<0.001
Parity	Nulliparous	1.00 (Reference)	-	1.00 (Reference)	-
	Multiparous	1.52 (1.03-2.25)	0.036	2.05 (1.46-2.88)	<0.001
Hypertensive Disorders	No	1.00 (Reference)	-	1.00 (Reference)	-
	Yes	2.15 (1.42-3.25)	<0.001	1.32 (0.91-1.92)	0.142
Fetal Distress	No	1.00 (Reference)	-	1.00 (Reference)	-
	Yes	3.02 (2.01-4.54)	<0.001	1.25 (0.85-.84)	0.256
Fetal Presentation	Cephalic	1.00 (Reference)	-	1.00 (Reference)	-
	Breech	1.85 (1.19-2.88)	0.006	2.98 (2.06-4.31)	<0.001
